# Comparative Insights to Advance Political Economy Analysis: A Response to Recent Commentaries

**DOI:** 10.34172/ijhpm.2023.8367

**Published:** 2023-12-13

**Authors:** Maria Nannini, Mario Biggeri, Giovanni Putoto

**Affiliations:** ^1^RCO (Action Research for CO-development), PIN Educational and Scientific Services for the University of Florence, Prato, Italy; ^2^Department of Economics and Management, University of Florence, Florence, Italy; ^3^Doctors with Africa CUAMM, Padova, Italy

 We are pleased to advance the debate on health coverage and financial protection in Uganda through a political economy perspective. Firstly, we express gratitude to the authors of the commentaries pertaining to our article “Health Coverage and Financial Protection in Uganda: A Political Economy Perspective.”^1^ These commentaries serve to draw attention to methodological aspects as well as context-specific political dynamics that impact the outcomes of the analysis. In this correspondence, we present key points from each commentary that we believe facilitate advancing the debate and catalysing a thoughtful analysis on health coverage and financial protection.

 Basaza and colleagues^2^ delve into the analysis of political economy, focusing on the introduction of the National Health Insurance Scheme (NHIS) in Uganda. The trajectory for implementing this scheme represents a distinctly political process and should be understood as part of the country’s long-term strategy for development; indeed, the NHIS is part of the national agenda “the Uganda Vision 2040.”^3^ Moreover, the government has officially committed to achieving the Sustainable Development Goals (SDGs), and there exists a roadmap aimed at attaining universal health coverage (UHC) in Uganda.^4^ As argued by the authors, political elites play a crucial role in determining the timing of reform implementation, also based on the existing consensus regarding the need for structural changes among policy makers and general public. Therefore, it is important not to perceive the current government position as permanent. Simultaneously, given the inherently political nature of the process, conducting interviews with political leaders belonging to the ruling party would enable the incorporation of central elements concerning interests and ideologies influencing the process. To facilitate the advancement of the discourse, it is beneficial to refer to recent data regarding misconceptions and fears. As articulated by the authors, the difficulty in mobilizing the informal sector to collect insurance premiums, as well as the low level of willingness to pay among the population, do not appear to genuinely hinder the design of the reform. In light of these new insights, it would be valuable to conduct a careful and updated analysis of the reform path to implement the NHIS, considering both the voice from the political elite as well as other important stakeholders.

 Croke^5^ directs attention to two elements that could strengthen the analysis of political economy, offering a clearer understanding of the dynamics characterizing the Ugandan case study. Firstly, the author suggests integrating the analysis with evidence from comparative politics focused on the current Ugandan regime. It is particularly useful to consider that, as concluded by various studies,^6-9^ the highly personalized nature of the regime in Uganda, centred around President Museveni, constitutes a significant aspect influencing choices related to priority reforms and allocation of budget across different sectors. Consequently, drawing from comparative politics literature, greater emphasis could be placed on the interplay between regime dynamics and state capacity. Secondly, the commentary delves into the concept of “political settlement” understood as “*the social order based on political compromises between powerful groups in society that sets the context for institutional and other policies.*”^10^ Referring to specific studies on the healthcare sector in Uganda,^11^ it is crucial to consider the role of political bargaining among influential actors and how it shapes health services delivery. These considerations also allow for advancing the specific discourse on the NHIS and the future prospects for healthcare sector financing.

 Eusebio and colleagues^12^ build upon our political economy analysis to delve into the pro-UHC forces that can expedite the political discourse and implementation of structural reforms in the healthcare sector. Firstly, the authors argue for the potential of reframing the UHC debate in order to catalyse effective change. They consider positioning UHC as a component of “nation-building” as an instrumental strategy to solidify a national identity and foster economic growth. Secondly, the commentary emphasizes the necessity of identifying or creating a window of political opportunity for health sector reforms, to be seized by relevant stakeholders. This could encompass leveraging the United Nations’ 2030 Agenda; harnessing the historical momentum generated by establishing a COVID-19 preparedness and response plan represents an additional opportunity to stimulate reforms for health coverage. Thirdly, the authors underscore the pivotal issue of mobilizing resources to drive meaningful change. They highlight the potential for progress through enhancing both the ownership of local communities in health financing programs and advocacy efforts by academic experts and civil society representatives aimed at overcoming existing barriers to UHC. Given the pluralistic nature of the Ugandan healthcare system, the political process should be open to engagement with all stakeholders, including, for instance, the Private Not-For-Profit sector.

 Fox proposes several potential methodological and theoretical improvements to enable deeper claims through political economy studies of health reforms in low- and middle-income countries. Firstly, the author underscores the significance of historicizing analyses. Taking into account past policies and the historical trajectory within specific contexts allows for easier identification of potential political decisions and structural reforms proposed by governments, considering the so-called policy feedbacks effects. Secondly, Fox suggests, whenever feasible, adopting a comparative perspective that examines various case studies aiming to move beyond describing a single case: indeed, more observations of different case studies would facilitate providing more generalized explanations of political economy dynamics according to a causal logic. Lastly, the commentary advocates for testing theories by verifying whether evidence supports or contradicts the expectations derived from the theory. This approach aims to avoid relying solely on a theory-light approach that risks not reaching meaningful conclusions to advance policy or practice.

 Kim^13^ applies a political economy approach to the case study of the Republic of Korea, identifying the most significant elements driving the reform process toward achieving UHC. The author traces the historical path followed by the country regarding its National Health Insurance system, identifying the primary obstacles hindering the actual attainment of UHC-related objectives. It is important to note that the analysis considers a relatively extensive period of reforms, starting from the 1960s, intending to present a more historically contextualized and in-depth study of the health insurance scheme.

 Tangcharoensathien and colleagues^14^ offer additional insights into the political economy processes aimed at achieving UHC in African low-income countries. Initially, the authors suggest a concise review of the available evidence concerning the impact of the pandemic and the recovery efforts in these nations. Secondly, they pinpoint the primary factors that adversely influence the attainment of health-related SDGs. Finally, the commentary highlights opportunities and provides recommendations for low-income country governments to implement effective reforms for UHC.

 Ssennyonjo^15^ delves into various aspects of analysis and suggests methodological enhancements to delve deeper into key concepts of political economy, thereby offering a better understanding of the ongoing dynamics in Uganda. Firstly, the author presents the “structure-agency” debate, emphasizing the crucial bidirectional interaction between structure and agency to enhance political economy analysis. Secondly, the commentary proposes additional considerations and existing theories to avoid oversimplifications concerning the meaning of ideas, interests, and institutions. For instance, when referring to “institutions,” formal or informal rules shaping or impacting human action^16^ should be considered.

 In conclusion, the analytical framework proposed in the article could benefit from several enhancements. Further to adopting terminologies and definitions more aligned with political economy analysis (for instance, rectifying the usage of the term “institutions”), embracing a more comparative approach represents a potential improvement. This approach would consider, on one hand, the overall architecture of different sectors and strategic priorities pursued by the most influential stakeholder groups in Uganda, thus disentangling the crucial aspect of trust and consensus towards political elites. On the other hand, it would encompass experiences from other countries regarding similar reforms in healthcare financing. Expanding the analysis in these two directions entails broadening the study’s focus, considering also a longer historical period to capture “policy feedback” effects. The broader scope is functional to understand the potential for change, overcoming existing barriers to achieve UHC.

## Ethical issues

 Not applicable.

## Competing interests

 Authors declare that they have no competing interests.
